# Analyzing the citation impact of predatory journals in the health sciences

**DOI:** 10.5195/jmla.2025.2024

**Published:** 2025-10-23

**Authors:** Erin Watson, Li Zhang

**Affiliations:** 1 e.watson@usask.ca, University of Saskatchewan Library, Saskatoon, Saskatchewan, Canada; 2 li.zhang@usask.ca, University of Saskatchewan Library, Saskatoon, Saskatchewan, Canada

**Keywords:** Predatory journals, citation analysis, health sciences, Web of Science

## Abstract

**Objective:**

Predatory journal articles do not undergo rigorous peer review and so their quality is potentially lower. Citing them disseminates the unreliable data they may contain and may undermine the integrity of science. Using citation analysis techniques, this study investigates the influence of predatory journals in the health sciences.

**Methods:**

The twenty-six journals in the “Medical Sciences” category of a known predatory publisher were selected. The number of articles published by these journals was recorded based on the information from their websites. The “Cited References” search function in Web of Science was used to retrieve citation data for these journals.

**Results:**

Of the 3,671 articles published in these predatory journals, 1,151 (31.4%) were cited at least once by 3,613 articles indexed in Web of Science. The number of articles that cited articles published in predatory journals increased significantly from 64 in 2014 to 665 in 2022, an increase of 10-fold in nine years. The citing articles were published by researchers from all over the world (from high-, middle-, and lower-income countries) and in the journals of traditional and open access publishers. Forty-three percent (1,560/3,613) of the citing articles were supported by research funds.

**Conclusions:**

The content from articles published in predatory journals has infiltrated reputable health sciences journals to a substantial extent. It is crucial to develop strategies to prevent citing such articles.

## INTRODUCTION

Predatory journals, which have been defined as “entities that prioritize self-interest at the expense of scholarship and are characterized by false or misleading information, deviation from best editorial and publication practices, a lack of transparency, and/or the use of aggressive and indiscriminate solicitation practices” [1], have become a concern in the health sciences [2]. Their lack of editorial rigour has consequences; articles published by such journals have been shown to be of lower quality than those published in reputable journals. Moher and colleagues found that only a small number of the articles published in predatory biomedical journals reported items such as ethics approval, funding source, blinding procedure, allocation methods, or, for systematic reviews, risk of bias assessment. As the authors state, these articles “consistently failed to report key information necessary for readers to assess, reproduce and build on the findings” [3]. Bianchini et al. found that the scores on PEDro (a critical appraisal tool used in physical therapy) received by randomized controlled trials published in journals on Beall's List (a list of potentially predatory journals) were significantly lower than those received by non-Beall's List journals [4]. And Nieminen and Uribe found that statistical methods were reported significantly less thoroughly by predatory dental journals than by non-predatory dental journals (whether they were open access or subscription-based) [5].

Citing articles published in predatory articles disseminates the unreliable data they may contain and may undermine the integrity of science. Although a significant number of studies have investigated the citation impact of articles published in predatory journals [6–11], relatively few have focused on health sciences. We will discuss these below.

Using data from Google Scholar, Nwagwu and Ojemeni studied 5,601 articles published in 32 Nigerian predatory biomedical journals. They found that, on average, each predatory journal received 394 citations, and each article published in these journals was cited 2.25 times [12]. Shamsi et al. also used Google Scholar data when investigating predatory dermatology journals, and found that of 4,164 articles, 1,146 appeared in Google Scholar, where they received on average, 4.1 citations each [13].

Oermann et al. found a much lower citation rate when they used data from Scopus to examine seven predatory nursing journals [14]. They found that these articles were cited 814 times by 141 non-predatory journals, and that each of the 7 predatory journals received, on average, 116 citations, with each of the non-predatory journals citing a median of two predatory articles. When examining citation data from Dimensions for 591 articles written by German researchers and published in 88 predatory medical journals, Stephen found that these articles were much more highly cited; they received on average 4.6 citations, nearly all from non-predatory journals. She concluded that predatory articles were extensively cited [15]. Dodgson et al. used a different approach; they investigated how many of the 127 manuscripts accepted for publication in the *Journal of Human Lactation* (a legitimate journal) from 2019-2021 cited articles published in predatory journals [16]. They found that 23 (18%) of these articles cited predatory articles.

While the extent to which predatory articles are cited varies from study to study, these studies demonstrate that predatory journal articles in health sciences have been cited, potentially polluting the scholarly record.

Citation of predatory journals by any type of article is problematic, but a concern particular to the health sciences is the inclusion of predatory articles in systematic reviews. Health care practitioners depend on systematic reviews and other knowledge syntheses to make informed clinical decisions. This means that including poor-quality articles in these reviews could threaten patient safety. Again, the extent to which this has occurred has been found to vary depending on the sample used. Ross-White et al. found that of a sample of 6,302 predatory journal articles, 120 were cited by 157 systematic reviews, of which 137 were published in reputable journals [17]. Collom et al. analyzed 78 review articles that cited predatory journals and found that 65.4% used these sources in a substantive way—either by including them in the review or by using them to support or extend the review's findings [18]. Notably, 39.2% of these reviews had a clinical focus. In contrast, Boulos et al. found that, of the 6,750 studies included in the systematic reviews published by two Cochrane networks in 2018 and 2019, only 55 were deemed to have been published in “questionable”, “likely predatory” or “presumed predatory” journals [19].

Research has also been done to determine who cites predatory journal articles. Some researchers have found that authors who cited predatory journal articles were primarily inexperienced researchers from Africa and Asia [6,20], although Oermann found that in nursing, most authors who cited predatory journals were American, Australian or Swedish [21].

As can be seen above, previous studies of citations of predatory health sciences journals have focused on particular aspects (such as a particular health sciences discipline, or authors or predatory journals from a particular country) of this topic. This study aims to take a broader approach by studying citations to articles published in a sample of predatory journals from across the health sciences.

Specifically, two questions will be addressed:

1. How often are articles published in predatory health sciences journals cited by articles published in legitimate journals?
2. What are the characteristics of legitimate articles and journals that cite predatory health sciences journals?

## METHODS

### Identification of Predatory Journals

To identify potential predatory journals, we went to the home page (https://www.omicsonline.org) of a publisher recognized as predatory by the US Federal Trade Commission [22]. From the “Journals” drop-down menu at the top of the page, we chose “Browse by Subject” and then in the “Journals by Subject” box, clicked the “Medical Sciences” category, which led to this web page: www.omicsonline.org/medical-sciences-journals.php. This page listed 26 journals in the Medical Sciences category; these were selected as the study objects for this research. To confirm that these journals were indeed predatory, we examined the website of each journal for common characteristics of predatory journals, such as false Journal Impact Factors or Cite Scores, misleading metrics (e.g., Index Copernicus), promotion of rapid publication, or obvious errors.

We recorded the first publication year and the most recent publication year for each journal. We manually counted the number of articles published by each journal, including only the document types (as identified by the journals) in which research was formally reported (research article/research, review, mini review, case report/study/series, brief report, market analysis, survey report, clinical investigation, and investigating article). We therefore excluded document types such as editorial, commentary, short communication, abstract only, book review, non-citable items (e.g., conference and award announcements), and articles that were no longer available on the website. A complete list of the journal titles can be found in the Appendix.

### Searching for Citations of Articles Published in Predatory Journals

The predatory journals we investigated were not indexed by Web of Science. Therefore, we searched the “Cited Work” (i.e., journal title) field using the “Cited References” search function in the Web of Science Core Collection; this allowed us to identify cited articles from the predatory journals, even though these articles were not indexed in this database.

Because journal titles are often abbreviated in Web of Science citation records, we used two methods to search for each title. First, we searched the truncated form of the journal title. For example, for the *Journal of Palliative Care & Medicine*, we searched “j* pall* care med*” in the Cited Work field. However, for journals that contained words commonly used in titles of health sciences journals (e.g., *Archives of Medicine*), searches using truncation retrieved too many results, making it unfeasible to review all the results. Therefore, we decided that if the truncated journal title search produced more than 5,000 results, we would search the exact journal title with quotation marks. Web of Science allows a maximum of 5,000 records to be downloaded when using the Cited References search function, making this a useful upper limit.

Because the Cited Work field in Web of Science retrieves articles from similar journal titles (e.g., *Journal of Preventative Medicine* vs. *Journal of Preventative Medicine and Hygiene*), we reviewed the result list and selected only the articles from the journals we were interested in. Then we downloaded the records for the articles published in predatory journals (subsequently referred to as “Predatory Articles”) and the records for the articles that cited the predatory articles (subsequently referred to as “Citing Articles”) for each journal.

The downloaded records of the predatory articles from Web of Science included many duplicate articles because if the original article was cited slightly differently (e.g., uppercase vs. lower case used in title, different version of author name, full journal title vs. abbreviation) by two different articles, it would show up in Web of Science as two different original articles. Therefore, we manually examined various fields in the downloaded records including article title, journal title, author, volume, issue, and page numbers for matches and, when necessary, consulted the website of the journal to confirm that the cited articles were the same. We merged the duplicates and their associated citation counts to obtain an accurate number of unique predatory articles. Data collection was completed in July 2023.

To identify the document types of citing articles, we searched the article titles for the words “systematic review”, “scoping review,” “meta analysis”, “meta-analysis”, “metaanalysis”, “realist review”, “randomized”, “randomised”, “guideline” and “guidelines”. We also scanned the titles to ensure that the results were actually publications of the desired types, rather than merely articles that discussed these document types.

## RESULTS

Of the 26 journals categorized as “Medical Sciences” on the predatory publisher website, 25 were published in English while 1 was published in Spanish. One journal (*Journal of Liver: Disease & Transplantation*) was subscription-based from 2012 to 2018 and only became fully open access from 2019, therefore, we excluded this journal. Thus, 25 journals were included in this study.

Of the 25 journals, most began publishing between 2015-2017, with the earliest started in 2005 (*Archives of Medicine*). At the time of data collection, 5 journals were inactive, while 20 were still publishing.

All 25 journal websites possessed characteristics of predatory journals. Most displayed a false journal impact factor and/or a false CiteScore. Some also indicated that they had a fast publication process or provided misleading information such as the Index Copernicus value or an incorrect definition of the *h-index*.

### Number of Predatory Articles

**Figure 1:**
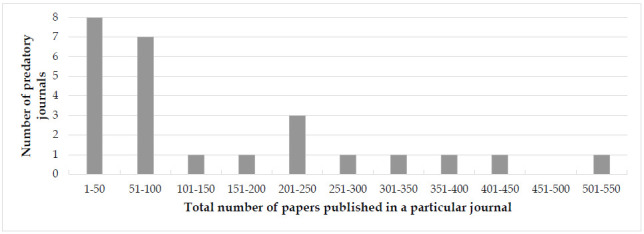
Distribution of number of papers published in predatory journals.

**Figure 2:**
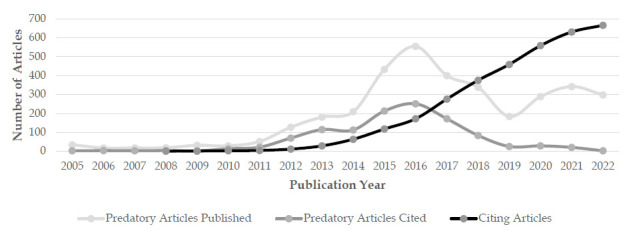
Trends in the number of predatory articles published, predatory articles cited, and citing articles.

Through manual counting, we identified 3,671 articles published in the 25 predatory journals. The average number of articles per journal was 147, though this ranged from 17 to 548. Fifteen journals published fewer than 100 articles (Figure 1). The total number of articles published per year in these journals began to increase in 2012 (when 127 were published), reached a peak in 2016 (when 544 articles were published), and then started to decline gradually with only 298 articles published in 2022 (Figure 2).

### Number of Predatory Articles Cited

The initial search of the full or truncated title of the 25 predatory journals in the “Cited Work” field within Web of Science found that 3,109 predatory articles were cited by Web of Science-indexed journals. As described in the Methods section, this set of articles included many duplicates. After merging the duplicates, we found that there were 1,151 unique predatory articles. Thus, out of the citable pool of 3,671 predatory articles, 31.4% (n=1,151/3671) were cited by Web of Science-indexed journals. On average, 27% of the articles in each of the 25 journals were cited in Web of Science, but this ranged from 1% (n=1/76 citable articles in *Journal of Preventive Medicine*) to 55.5% (n=15/27 citable articles in *Evidence Based Medicine and Practice*).

While the number of predatory articles cited remained fairly low from 2005 to 2010, it started to grow in 2011 and reached its peak in 2016 (Figure 2). About half (n=574/1,151; 49.9%) of the cited articles were cited once and about half (n=577/1,151; 50.1%) more than once. Seven (0.6%) articles were cited more than 30 times (Table 1).

**Table 1:** Number of predatory articles by times cited.

Times cited	Number of articles
1	574
2	234
3	108
4	61
5	45
6-9	70
10-19	43
20-29	9
≥30	7

### Number of Citing Articles

We identified 3,613 articles that cited 1,151 predatory articles, so each article was cited on average 3.14 times.

Figure 2 also shows a comparison of the number of predatory articles published to the number of citing articles each year. From 2014 on, the number of citing articles increased steadily. There were 64 citing articles in 2014 and 665 citing articles in 2022, an increase of 10-fold in 9 years. The Mann-Kendall trend test and Sen's slope test were performed in R, and the results showed that the increasing trend in Citing Articles is significant from 2008 to 2022 (z=5.1467, p-value<0.00001, Sen's slope =53.5).

**Figure 3:**
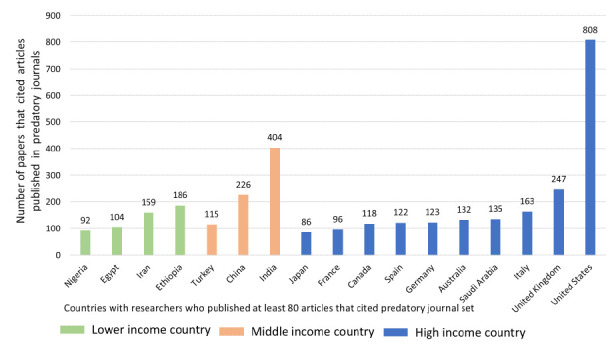
Citing articles by country income level for countries with at least 80 citing articles.

Note: Country classification by income level provided by World Bank.

Predatory journal articles were cited by researchers from high-income countries such as the United States and the United Kingdom, as well as by researchers from middle-income and lower-income countries (Figure 3).

### Characteristics of Citing Articles

The 3,613 citing articles appeared in journals published by both traditional commercial publishers such as Elsevier and Springer, and newer, open access publishers such as MDPI (Table 2). These three publishers contributed 543 (15%), 323 (8.9%), and 254 (7.0%) citing articles respectively.

**Table 2:** Publishers with at least 50 citing articles

Publisher^1^	Number of Citing Articles
Elsevier	543
Springer	323
MDPI	254
Wiley	229
Taylor & Francis	163
Biomed Central	162
Sage Publications	134
Lippincott Williams and Wilkins	112
Hindawi	91
Wolters Kluwers Medknow	78
Oxford University Press	72
Frontiers Media	67
Total	2,228

Note:

1 Publisher names shown in this table are as reported by Web of Science. We did not include citing articles published by subsidiary publishers when reporting the numbers published by the parent company.

The majority of the 3,613 citing articles were categorized by Web of Science as being on the broad topic of Life Sciences & Biomedicine (n=2,921, 81%). Articles in other Categories (Technology, Social Sciences, Physical Sciences, and Arts & Humanities) composed only 19% (n=692) of the total 3,613 citing articles.

Detailed characteristics of citing articles can be found in Table 3. Sixty-nine percent (n=2,492/3,613) of the citing articles were published in journals indexed in Science Citation Index (SCI*),* and 17% (n=604/3,613) in Social Sciences Citation Index (SSCI), two of the most-used sources for journal quality assessment. When combining the two indexes, we found that 2,619/3,613 (72%) of the citing articles were published in journals indexed in either or both. We also noted that 37 (1%) of the 3,613 citing articles were considered by Web of Science to be “highly cited” (or in the top 1% of papers in their field by citation).

**Table 3:** Characteristics of citing articles.

	Indexed in				Has ORCID?^5^		Funded research?		Document Type		
	SCI^1^	SSCI^2^	ESCI^3^	Other^4^	Yes	No	Yes	No	Review	Article	Other Types
Number of citing articles*	2,492	604	885	140	2,606	1,007	1,560	2,053	767	2,624	222
Percentage*	69%	17%	24%	4%	72%	28%	43%	57%	21%	73%	6%

Note:

1 Science Citation Index;

2 Social Sciences Citation Index;

3 Emerging Sources Citation Index;

4 Other includes Arts & Humanities Citation Index, Book Citation Index, and Conference Proceedings Citation Index;

5 At least one author provided an ORCID ID.

* In Web of Science, a journal may be included in multiple citation indexes. As a result, the total number of citing articles across all citation index categories exceeds the actual number of citing articles (3,613). This also means that the sum of percentages for all indexes is greater than 100%.

Of the 3,613 citing articles, 1,560 (43%) listed a funding source. Of this funded research, 310 (19.9%) studies were funded by 11 major funding bodies for health research (National Institutes of Health, Wellcome Trust, European Commission, European Research Council, U.K. Medical Research Council, France's Institut national de la santé, U.S. Department of Defense, Canadian Institutes of Health Research, Australian National Health and Medical Research Council, Howard Hughes Medical Institute and Deutsche Forschungsgemeinschaft).

In terms of publication types, of the 3,613 citing articles, 767 (21.2%) were review articles. The vast majority (n=2,624; 72.6%) were non-review journal articles (and were not letters or editorial material). There were 7 (0.2%) practice guidelines, 137 (3.8%) systematic reviews, 83 (2.3%) meta-analyses (of which 61 (1.7%) were also labelled as systematic reviews), 32 (0.9%) scoping reviews, and 1 (0.03%) realist review among the citing articles. There were also 40 (1%) randomized controlled trials.

## DISCUSSION

In this study, we analyzed the characteristics of articles published in 25 predatory journals and the articles that cited them. We found that the number of articles published per year by the predatory journals in our sample grew from 2011 until 2016, and then began to decline. This was likely due to increased awareness of predatory journals among researchers in the health sciences. Oermann et al. found that by 2021, 631 articles on predatory publishing in health care had been published, many of which warned researchers about the hazards of publishing in predatory journals [2].

The number of predatory articles cited began to increase in 2010 and peaked in 2016. There are two likely reasons for the decrease after 2016. First, as noted above, the number of articles published in this predatory journal article sample started to decline in 2016, so the number of articles available to be cited is smaller, which could have led to a smaller number of citations. Second, articles are often not cited for three to five years after being published, and so it is possible that the articles in our sample that were published in more recent years have just not had enough time to be cited [23]. While the decline in citations could be the result of researchers' increased awareness of predatory journals (and their decision not to cite them), this seems unlikely given that the number of citing articles increased steadily from 2014 to 2022 (Figure 2).

Aside from the alarming finding that the number of citing articles soared 10-fold from 2014 to 2022, there are several other indications that the influence of predatory articles on scholarship in the health sciences has increased over time. First, we found that 31.4% of the predatory articles were cited by articles in journals indexed in Web of Science. This number is considerably higher than the rate of 6-13% reported in other studies which also used Web of Science citation data [8,20]. The previous studies looked at Turkish predatory journals (from a variety of disciplines) and predatory journals in the social sciences. Our much higher citation rate may indicate that in the health sciences, content from predatory journals has infiltrated reputable journals more extensively than has occurred in other fields. Further, about 72% of the citing articles in our study were published in journals indexed in the Science Citation Index or the Social Sciences Citation Index (rather than the Emerging Sources Citation Index, an index used mainly for newer journals, or other Clarivate Citation Indexes), showing that most of these citations came from important, established journals, which may have a higher potential to spread the unreliable information contained in predatory articles. In addition, because Web of Science is known to be selective when choosing journals for indexing, if citations from all journals were included, the total number and average number of citations received by our sample would likely be higher than what we reported here.

An additional item of concern is the continued evidence of incorporation of predatory articles into not just knowledge syntheses, but practice guidelines. Our study found that 7 practice guidelines, and 137 systematic reviews of which 61 were meta-analyses, and a further 22 meta-analyses not also described as systematic reviews, cited the predatory article sample.

While Akça et al., Frandsen, and Oermann et al. found that most of those who cited predatory journals were based in particular regions [6,20,21], our study's results were somewhat different. We found that the citing articles were authored by researchers all over the world, though those from the U.S., India, and the U.K. were the most likely to cite predatory articles. In addition, our results show that 43% of the research was funded (with 19.9% funded by 11 major funding bodies for health research) and that most papers were associated with at least one ORCID ID, showing that, contrary to what was found by Akça et al. and Frandsen, at least some authors of citing articles are experienced researchers. The largest number of citations of our sample came from journals published by the large, established publishers Elsevier and Springer, followed by MDPI, a major open access publisher. Citing predatory research, then is not something done only by inexperienced researchers or by small publishers.

Predatory journals are often unstable; they lack long-term archiving or preservation mandates, and their websites can be shut down or changed at any time. This instability poses challenges for those studying the effects of predatory journals. For instance, at the time of writing this manuscript, the URL for the *Journal of Gastrointestinal Cancer and Stromal Tumors*, one of the 25 predatory journals selected for our study, was redirecting to the site of another predatory journal, the *Journal of Cancer Science and Research*. However, when we searched Web of Science, we found citations for articles published under the title *Journal of Gastrointestinal Cancer and Stromal Tumors*. When we examined the full-text PDFs of the cited predatory articles, we found that some PDFs displayed the OMICS logo and the title *Journal of Gastrointestinal Cancer and Stromal Tumors*, while others showed both the titles *Journal of Gastrointestinal Cancer and Stromal Tumors* and *Journal of Cancer Science and Research*. This suggests that both journal titles might have been used for the same journal at some point to attract more submissions. We suggest that in future, those studying the citation impact of predatory journals save or take screenshots of their websites to allow readers to verify and reproduce the results.

Our study has a few limitations. First, as has been noted by others, citation of an article does not necessarily mean that the citing author agrees with, or approves of, the research done by the cited author. So, while it is possible that some of the predatory articles in our sample were cited for the purpose of criticizing them, it has been found that authors only rarely cite articles for negative reasons, so it seems likely that that would be the case in our sample as well [24]. Second, as indicated in the methods, some of the predatory journals had titles that made doing a comprehensive search for them very difficult. It is therefore possible that the number of citations received by articles published in these journals was even higher than indicated here. Conversely, following the practice of Web of Science [25], we counted only the number of citable items (e.g., research articles) published in the predatory journals but we did not check that the citations to these journals were always to the citable items that we had counted instead of to non-citable items such as editorials or other publication types. So, the number of citations per article reported in this study may be slightly inflated. Finally, similar to many other bibliometric analyses, our results rely on citation data from Web of Science. The accuracy of our findings depends on the precision of the data provided by this database. However, several studies have shown that citation database data can sometimes be inaccurate due to citation errors by authors or mistakes made by databases during data entry or within their internal citation matching algorithms [26,27]. Nevertheless, our study provides a snapshot of how articles published in predatory health sciences journals have been cited by other works.

Citing predatory articles lends them an air of legitimacy and respectability, thus making them an even more attractive place to publish [28]. If authors continue to cite predatory articles, what is the solution?

The International Committee of Medical Journal Editors (ICMJE), in its “Recommendations for the Conduct, Reporting, Editing, and Publication of Scholarly work in Medical Journals”, advises (and has done so since 2019) that “Authors should avoid citing articles in predatory or pseudo-journals” [29]. Similarly, the Committee on Publication Ethics (COPE) states in its discussion document on predatory publishing that authors, professional societies and institutions should “avoid citing predatory journal articles and beware when performing systematic and meta-analyses” and that reviewers and editors, journals and publishers, funders and institutions should all “discourage” citations of articles from predatory journals [30]. So, it is not for lack of guidance that authors are citing predatory articles.

During the last decade, there have been considerable efforts to promote the awareness of characteristics of predatory journals among researchers and provide them with strategies to avoid publishing in these venues. While these strategies seem to have been successful (as shown in our study by the declining number of predatory articles), it is time to develop strategies to prevent citation of predatory journals. Authors, editors, publishers, and peer-reviewers all have a role to play in curbing the influence of predatory journals and in protecting the integrity of science [31]. Part of the solution may lie in non-predatory journals changing their own policies. If such journals adopt open peer review and institute data sharing policies, require trial registration and adherence to reporting standards, and for systematic reviews, require risk of bias assessment, it will make it easier for researchers to identify and avoid citing predatory journals, which presumably, would not have such policies [28]. Publishers and/or editors could also ask authors to confirm, when submitting a manuscript, that they have not cited predatory journals [10]). As the ICMJE has stated, we must “avoid engaging these charlatans….to strengthen and preserve the trust that is central to science and medicine” [32].

## Data Availability

Data associated with this article are available in the Federated Research Data Repository at: DOI: https://doi.org/10.20383/103.0998
