# Supplementary material for: Analyzing the citation impact of predatory journals in the health sciences
Source: J Med Libr Assoc. 2025 Oct 23;113(4):327–35. doi: 10.5195/jmla.2025.2024 (PMC12606076; doi:10.5195/jmla.2025.2024)
Supplement: Supplementary file 1 — Appendix A [file jmla-113-4-327-s01.pdf]

Appendix A
List of 26 Journal Titles

Journal of Palliative Care & Medicine
Translational Biomedicine
Journal of Gastrointestinal & Digestive System
Journal of Biomedical Sciences
Archives of Medicine
OMICS Journal of Radiology
Journal of Regenerative Medicine
Occupational Medicine & Health Affairs
Journal of Preventive Medicine
Herbal Medicine: Open Access
Journal of Imaging and Interventional Radiology
Archivos de Medicina
Journal of Medical Physics and Applied Sciences
Journal of Gastrointestinal Cancer and Stromal Tumors
Journal of Medical Implants & Surgery
Medical & Clinical Reviews
Pediatric Emergency Care and Medicine: Open Access
Insights in Biomedicine
Evidence based medicine and practice
Journal of Blood Research & Hematologic Diseases
Medical case reports
Journal of Medical Toxicology and Clinical Forensic Medicine
Journal of Bone Reports & Recommendations
Journal of Hepatitis
Journal of Cancer Diagnosis
